# Palpitations in puerperium—a self-recorded smart watch ECG gives the hint to hormone-induced ventricular arrhythmia: case report

**DOI:** 10.1093/ehjcr/ytae166

**Published:** 2024-04-02

**Authors:** Paulina Anna Jankowska, Christian Georgi, Marwin Bannehr, Christian Butter

**Affiliations:** Department of Cardiology, Heart Center Brandenburg, Brandenburg Medical School (MHB) Theodor Fontane, Germany; Department of Cardiology, Heart Center Brandenburg, Brandenburg Medical School (MHB) Theodor Fontane, Germany; Department of Cardiology, Heart Center Brandenburg, Brandenburg Medical School (MHB) Theodor Fontane, Germany; Department of Cardiology, Heart Center Brandenburg, Brandenburg Medical School (MHB) Theodor Fontane, Germany

**Keywords:** Ventricular tachycardia, Wearables ICD, Rhythm detection, Gender medicine

## Abstract

**Background:**

Gender-related aspects in cardiac arrhythmias have gained increasing attention, still the understanding of peripartum electrical disorders remains vague.

**Case summary:**

A 28-year-old woman developed palpitations and presyncopes during the post-partum period after her second pregnancy. Palpitations remained unclear until a self-recorded single-lead smartwatch ECG revealed a complete episode of a fast broad complex tachycardia (260 b.p.m.) that led to hospital admission. Echocardiography, cardiac magnetic resonance imaging, and exercise testing, showed no relevant abnormalities. Recording the tachycardia in a 12-lead-ECG could eventually be achieved revealing an inferior axis and positive concordance in the precordial leads. Episodes of ventricular tachycardia (VT) could be provoked by breast feeding and mental stress, but not induced in two electrophysiological studies. Genetic testing was normal. The patient continued to experience repeated, self-terminating VT episodes, lasting between 10 and 40 s, leading to presyncopes and a syncope with a fall. The beginning of symptoms subsequent to child birth and frequent premature ventricular contractions in her first pregnancy made hormone-induced arrhythmia a tentative diagnosis. Heart rate-corrected QT (QTc) intervals showed significant variability corresponding to the frequency of episodes in a retrospective evaluation. The cessation of breastfeeding led to a termination of arrhythmias. The patient was temporarily equipped with a wearable cardioverter defibrillator vest, an implantable cardioverter defibrillator (ICD) was not implanted.

**Discussion:**

The case report highlights the potential of self-recorded, patient-activated ECG monitoring in diagnosing recurrent palpitations, and the dilemma of timing for implanting ICDs in young patients with ventricular arrythmias (VTs). Additionally, it underlines the role of post-partum hormones in the susceptibility to ventricular arrhythmias, calling for further research of gender-specific, and pregnancy-associated arrhythmias.

**Learning points**

- The influence of sex hormones on arrhythmia needs further research and should not be neglected in differential diagnosis
- The self-recorded, patient-activated ECG monitoring may be crucial for finding the right diagnosis and treatment as soon as possible

## Introduction

Gender differences in the prevalence of certain arrhythmias suggest a correlation between sex hormones and the risk of developing arrhythmias.^1–4^ Hormonal influence on ion channel function can cause alterations in the action potential in cardiac myocytes leading to changes in the heart rate-corrected QT (QTc) interval, especially in channelopathies like long QT syndrome, Brugada syndrome, and catecholaminergic polymorphic ventricular tachycardia.^5^ Prolactin and oxytocin hormones can also affect the regulation of transcriptional processes. The underlying mechanisms are not yet fully understood, which makes treatment difficult.^6^ The use of new patient- and symptom-oriented rhythm monitoring methods has the potential to improve the early detection of arrhythmias.

## Summary figure

**Figure:**
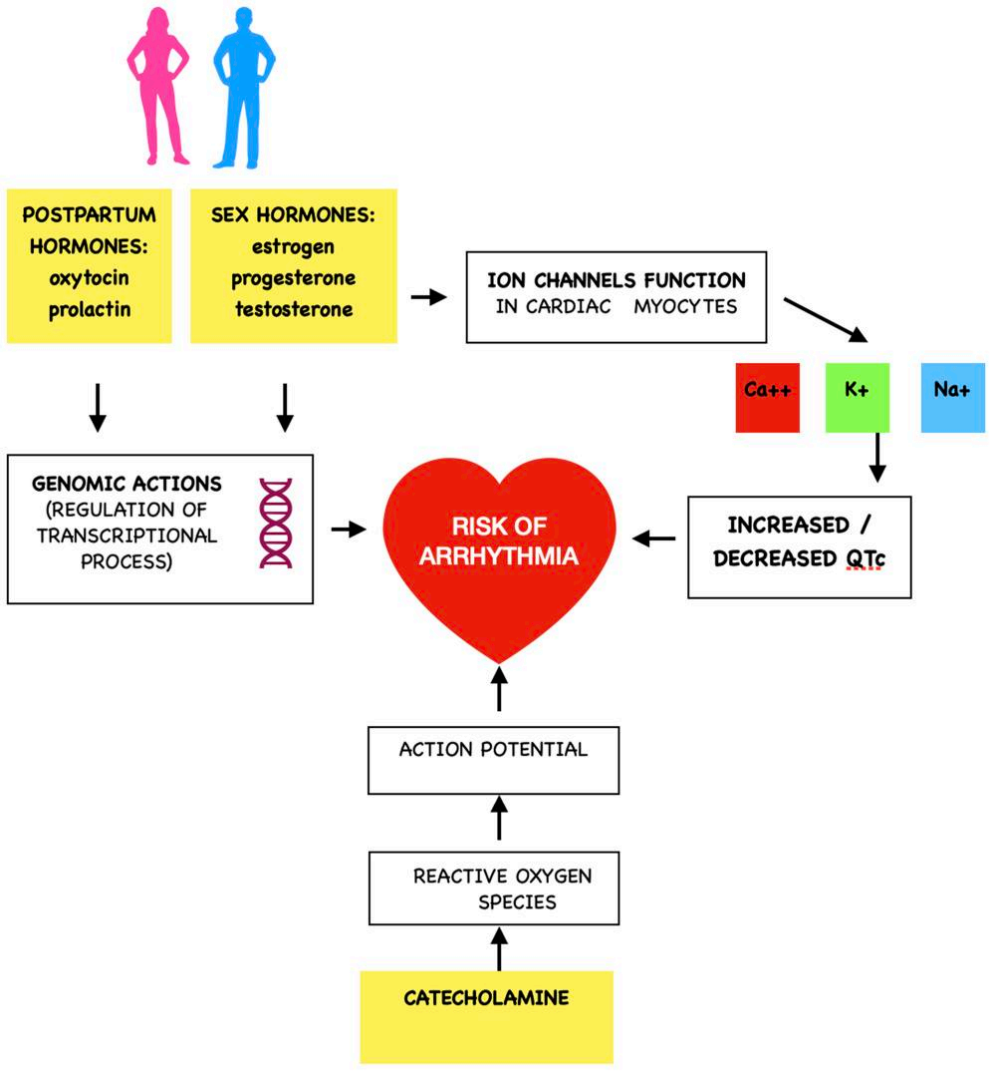
Association of sex hormones and arrhythmias.

## Case presentation

A 28-year-old woman without pre-existing conditions was admitted to the emergency department with repeating presyncopes. She had given birth three weeks before to a healthy daughter, and 3 years before to a healthy boy. She already suffered from frequent premature ventricular contractions (PVCs) during her first pregnancy. Family history did not reveal any hint of genetic heart disease or sudden cardiac death.

**Figure 1:**
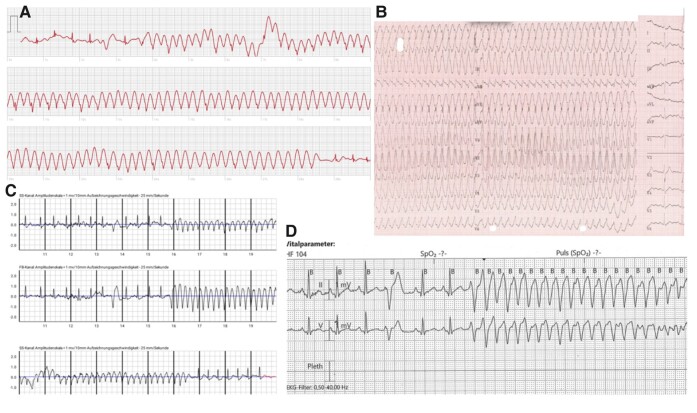
Different forms of rhythm monitoring showing the clinical ventricular tachycardia. (*A*) SmartWatch documentation, (*B*) 12-lead-ECG, (*C*) wearable cardioverter defibrillator recording, and (*D*) in-hospital telemetric monitoring.

**Figure 2:**
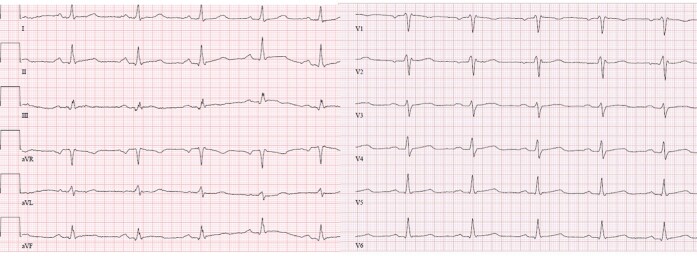
The resting 12-lead-ECG from the first hospital admission.

The patient, a nurse herself, initially assumed she had orthostatic vertigo. As the symptoms increased, she succeeded to record an episode of tachycardia on her SmartWatch (*Figure 1*) during one of the episodes while shopping. The automatic analysis revealed suspicion of ventricular tachycardia (VT), so the woman sought medical help immediately. After admission, telemetry monitoring revealed further episodes of non-sustained but fast and highly symptomatic, monomorphic VTs, especially during emotional stress and breastfeeding. Resting ECG (*Figure 2*) and treadmill stress test did not reveal any pathology. Echocardiographic left ventricular ejection fraction (LVEF) was 68%, TAPSE 21 mm, and dimensions of chambers were normal. Cardiac magnetic resonance imaging (cMRI) showed no evidence of oedema, fibrosis, or late enhancement.

In an electrophysiologic (EP) study, programmed atrial stimulation induced atrioventricular nodal reentrant tachycardia——without correlation to the clinical symptoms. VTs or frequent PVCs were absent despite aggressive programmed ventricular pacing manoeuvres. Given a slight clinical improvement under beta-blocker therapy but without clear diagnosis, the young mother urged discharge.

Eleven days later, the patient was readmitted to the emergency department with significantly increased symptoms of up to 10 presyncopes per day and frequent palpitations. Telemetric monitoring showed the previously seen VTs and a clear ECG-symptom-correlation. A second EP test with high-resolution 3D-endocardial mapping of the left ventricle failed to identify a suitable ablation target due to a lack of spontaneous or provoked ventricular arrhythmia. Rare PVCs suggested a left anterolateral epicardial focal origin close to the mitral valve annulus, as indicated in the 12-lead-ECG. An ablation attempt via the coronary sinus was not successful, and an epicardial approach was not pursued due to low periinterventional PVC burden and higher complication risks.

The patient was discharged again on her request, this time equipped with a wearable cardioverter defibrillator vest (WCD). Two weeks later the young mother collapsed while walking with her baby. As she regained consciousness, the baby next to her on the ground after falling out of the stroller, she was able to suppress WCD therapy. The recorded WCD ECGs and telemetry revealed an increased number of PVCs and non-sustained ventricular tachykardias (nsVTs), especially during breastfeeding.

**Figure 3:**
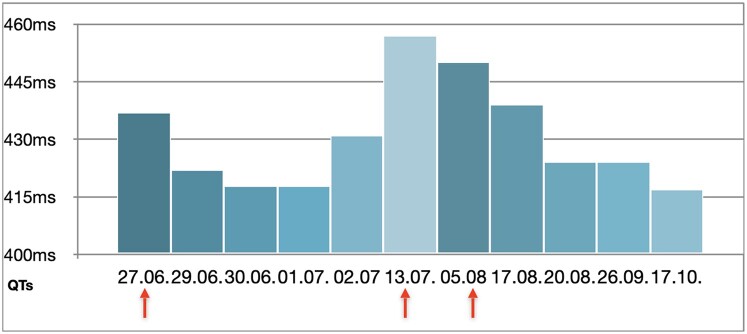
Correlation between symptoms and QTc interval prolongation in all of the patient’s documented ECGs; the days of hospital admissions are marked with arrows.

Analysis of all of the patient’s ECGs revealed a correlation between the severity of symptoms and an increase in QT intervals, with the longest QTc observed on admission days and a decrease under intensified beta-blocker therapy during hospitalizations (*Figure 3*).

The progressive nature of the symptoms, correlation between breastfeeding and tachycardia, absence of structural heart disease and significant QTc prolongation made hormone-induced VT the most likely diagnosis. Having no other therapy options and after weighing risks for mother and child, the patient could be convinced to cease breastfeeding. Clinical improvement was rapidly observed, with a reduction in PVCs and no further episodes of nsVTs. The patient was repeatedly seen in our outpatient clinic over the next 12 months, with no signs of palpitations, or manifest tachycardia. The QTc interval remained constant. Considering possible side effects, the rapid improvement of symptoms after trigger elimination, negative genetics (panel of genetic testing for commonly affected genes in inherited channelopathies/cardiomyopathies genes: CACNA1C, CALM1, CALM2, CALM3, CASQ2, DSG2, DSP, DSG2, DSP, JUP, KCNE1, KCNE2, KCNJ2, RYR2, SCN5A, ABCC9, AKAP9, ANK2, CACNA2D1, CACNB2, CAV3, CDH2, CTNNA2, DES, FLNC, GATA5, GATA6, GJA5, GPD1L, HCN4, KCNA5, KCND3, KCNE5, KCNJ8, KCNQ1, LDB3, LMNA, PKP2, PLP, PPA2, PPKAG2, RAMGRF, SCN10A, SCN2B, SCN3B, SLMAP, SNTA1, TBX5, TECRL, TGFB3, TMEM43, TNNI3, TNNI3K, TNNT2, TRDN, TRPM4, TTN) and patient’s wish we refrained from implantable cardioverter defibrillator (ICD) implantation, although guideline accordance recommending an ICD for secondary prevention in a sustained symptomatic VT with subsequent syncope.

## Discussion

This case of a young mother facing life-threatening arrhythmias highlights several important issues in the management of VTs (*Figure 2*).

Firstly, it emphasizes the value of self-recorded, patient-activated ECG monitoring in recurrent palpitations and presyncopal situations.^7^ Without it the symptoms might have been misinterpreted and trivialized by hospital staff and the patient herself.

Secondly, it raises the question of timing in ICD implantation in young patients.^8^ What is the greater harm? Implanting a young patient an allegedly dispensable transvenous or subcutaneous ICD foreseeing future side effects as lead complications, pocket infections or inadequate therapies or putting a young mother at risk by observing multiple episodes of VTs without having a clear cause? In our case, the constantly self-terminating nature of VTs, patient’s preference and successful trigger elimination made us decide against an ICD implantation.

Lastly, it underscores the role of sex and post-partum hormones in the susceptibility to ventricular arrhythmias. Both oxytocin and prolactin levels increase post-partum and rise especially during breastfeeding,^9^ with prolonging effects on the action potential duration and QTc interval. Furthermore, oestrogen and progesterone levels rise in the first post-partum weeks, which may influence the function of ion channels and thus the action potential. Further research focused on sex-specific and pregnancy-associated arrhythmias will be necessary to gain deeper insights into gender-related aspects of ventricular tachycardias.

## Supplementary Material

ytae166_Supplementary_Data

## Data Availability

Data underlying this article are available in the article and in its online Supplementary material.

## Lead author biography

Paulina Jankowska—medical doctor during residency in cardiology in Heart Center Brandenburg. After graduating from Jagiellonian University Medical College in Cracow she chose to specialize in cardiology, main interest in electrophysiology. She is a member of German Cardiac Society.

## Supplementary material

**Patient consent:** Patient’s written consent has been obtained in accordance with the principles of COPE.

**Funding:** The authors receives no financial support for the research and the authorship. The publication of this article is probably going to be supported by Brandenburg Medical School (MHB) Theodor Fontane – we are still waiting for the final.
